# Biophilia in Italian preschool children: preliminary findings

**DOI:** 10.3389/fpsyg.2025.1567848

**Published:** 2025-04-09

**Authors:** Sabine Pirchio, Sara Costa, Rosa Ferri

**Affiliations:** ^1^Department of Dynamic and Clinical Psychology and Health Studies, Sapienza University of Rome, Rome, Italy; ^2^Department of Education, Roma Tre University, Rome, Italy

**Keywords:** biophilia, pro-environmental behaviors, nature connectedness, preschool children, interview

## Abstract

This study investigated the relationship between biophilia, pro-environmental behaviors, and connectedness to nature in Italian preschool children. A total of 196 children (ages 24–65 months) and their parents participated. Children’s biophilia and pro-environmental behaviors were assessed through a role-playing interview, while children connectedness to nature, experiences in the nature, and parents’ pro-environmental behaviors were measured through indirect measures (questionnaires). The interview scores revealed significant positive correlations with children’s connectedness to nature as well as nature exposure reported by parents even if not in every age group. Children’s nature connectedness also correlated with pro-environmental behaviors and marginally, with parents’ pro-environmental behaviors. The results showed that, as expected given the innate component of biophilia, biophilia scores were not significantly different across age groups, while older children engage in more pro-environmental behaviors than younger children, suggesting the significant role of education and socialization. These findings highlight the complexity of assessing preschool children’s biophilia and connectedness to nature, particularly when combining direct measures (child interviews) with indirect measures (parental reports) and underscores the need for further research to refine the conceptualization of these constructs and explore their developmental trajectories.

## Introduction

Following the work by Fromm (1973), and by Kellert and Wilson (1993) biophilia can be defined as the human natural tendency to be attracted to and focus upon life, including humanity and nature, and to emotionally affiliate with them. This tendency is genetically grounded in our evolution since Paleolithic and results in the development of a personality trait leading to the adoption of deliberated behaviors and responses when in contact with nature (Barbiero and Berto, 2021). The phylogenetic origin of biophilia rooting in late Pleistocene explains the preference for wilder natural environments (e.g., savannah) showed by people with higher affiliation with nature compared to people with lower affiliation with nature who would prefer more domestic natural environments. On the other hand, even if hereditary and manifest in young children’s behaviors and preferences, biophilia needs to be promoted by actual experiences with and in the nature (Barbiero and Berto, 2021).

A rich literature shows as the contact with nature presents benefits for human subjective well-being in adults (Hartig, 2004; Lafortezza et al., 2009; Hartig et al., 2011; Carrus et al., 2015b, 2017; Wassenberg et al., 2015), and a growing number of studies have shown the impact of the experience of restorative environments on children (e.g., Bagot et al., 2015; Carrus et al., 2015a; Collado and Staats, 2016; Pirchio et al., 2021). The qualities of life environments (e.g., rural *vs.* urban) may play a role in happiness and wellbeing of adults and children (Cerina and Fornara, 2011; Kabisch et al., 2017; Maricchiolo et al., 2020) through the action of environmental affordances and their interaction with the individuals’ behaviors, intentions and choices (Carrus et al., 2020).

Children in western post-industrialized countries living in urban areas spend most of their time indoor in their home environment, at school, and for leisure activity time (Marte et al., 2020; Pirchio et al., 2021). This limited access to nature in early years of life raises concerns for the child physical, cognitive, social, and ecological development (Louv, 2005) impoverishing the opportunities for children to benefit from the contact with nature for attention restoration (Kaplan and Berman, 2010; Johnson et al., 2019) and stress reduction (Carrus et al., 2015a).

In fact, contact with nature has been found to be linked with children’s wellbeing and cognitive performance, affective states, and social behavior (Carrus et al., 2015a; Federico, 2020; Harvey et al., 2020; Pirchio et al., 2021; Tarman et al., 2023).

Also, contact with nature may increase children’s biophilia and connectedness to nature which are important factors in the promotion of pro-environmental behaviors (Liefländer et al., 2013). Research has found how important are living environments designed to be as similar as possible to natural environments for psychological wellbeing (Berto et al., 2020). Also, a robust tradition of environmental education programs proposes to use experiences in nature to improve children’s connectedness to nature to produce an impact on their pro-environmental behavior (Otto and Pensini, 2017; Passafaro et al., 2010; Varela-Candamio et al., 2018).

Children’s connectedness to nature and pro-environmental behavior may also be importantly influenced by factors related to their parents, like general factors such as education level and family income, educational factors such as parenting style, and specific ecological factors such as nature experiences during their childhood, nature connectedness, and interest in nature (Wu et al., 2023) and the naturalness of the landscape in the place where they live (Barbiero and Berto, 2021; Stocco et al., 2023). However, studies in this area are mostly conducted using parents’ reports about their preschool child’s connectedness to nature and pro-environmental behaviors, and there are still a limited number of studies directly investigating this relationship by direct measures on children.

The aim of this study is to investigate the relationship of biophilia and pro-environmental behaviors measured in Italian preschool children with their connectedness to nature and their nature exposure reported by their parents, and with the parents’ pro-environmental behaviors. To this aim we adapted the Biophilia Interview (Rice and Torquati, 2013) that has been developed to measure the affiliation with nature of children aged 34–69 months living in California and Nebraska showing fairly high scores regardless of the naturalness of the preschool environment and of parents’ education and family income. The Biophilia Interview has been later adapted (with some changes in the materials and procedures) by Yilmaz (2017) and then used with Turkish children in relation with measures of social–emotional wellbeing, finding a relationship between self-control and biophilia (Tarman et al., 2023). The Biophilia Interview has also been adapted and used with 51 Swedish children between 3 and 5 years of age findings high scores consistent with teachers’ reports about children’s preferences and experiences with nature (Beery et al., 2024).

## Method

### Procedure

To gain access to preschools, the school principals were initially contacted and subsequently connected us with the educators. Once communication was established with the educators, informed consent forms were distributed to parents. After the signed consent forms were returned, the research activities began. Data collection took place in eight different preschools in a central region of Italy.

Appointments were scheduled with the educators, and on the agreed dates, interviews with the children were conducted in a quiet setting, separate from their classmates. The activity with children lasted about 10 min. The educators played a crucial role in facilitating communication with the children’s parents, as well as distributing and collecting the completed questionnaires.

The study was approved by the Ethics Committee of the Department of Dynamic and Clinic Psychology and Health Studies of Sapienza University of Rome (prot. num. 0000550), ensuring compliance with ethical research standards.

### Participants

The study included a total of 196 children (80 females) and one of their parents. The children had a mean age of 55 months (*SD* = 11.75; *range* = 24–65). Most of the parental questionnaires were completed by mothers (*n* = 155), compared to fathers (*n* = 19). The parents had a mean age of 37.5 years (*SD* = 4.8). Some parents do not have returned the questionnaire despite consenting to participate, leading to the exclusion from the final analysis of 22 children. Therefore, final sample consisted of 174 dyads.

### Measures

#### Biophilia

Children’s biophilia was assessed using a role-playing interview developed by Rice and Torquati (2013). This semi-structured interview utilized puppets to elicit responses from children, making the process engaging and age appropriate. To the original 11 items, 4 items were added to assess children’s pro-environmental behavior (e.g., “This boy (or girl, if the respondent was female) turns off the water while brushing their teeth”). The interview showed a good internal consistency (*α* = 0.76).

#### Connectedness to nature

To assess children’s connectedness with nature we used the Connectedness to nature index—parents of preschool children (CNI-PPC) by Sobko et al. (2018). The questionnaire consists in 16 items to captured children’s enjoyment of nature, empathy for nature, responsibility toward nature, and awareness of nature. Internal consistency was high (*α* = 0.92).

#### Parents’ pro-environmental behavior

We used the Italian General Ecological Behaviour Questionnaire (Kumawat and Pronello, 2021) to assess the pro-environmental behaviors of parents. The questionnaire consists of 26-item to assess the pro-environment behavior in everyday life. Cronbach’s alpha is 0.83.

#### Nature exposure

To assess children’s engagement with natural environments we asked frequency of visits to specific outdoor locations, excluding school-related activities. Parents were asked to recall how often their child visited four types of locations with natural elements during the last month: public parks, beaches, countryside natural areas, and mountain natural areas. The frequency was measured using a 5-point scale from “never” to “more than 3 times per week.”

### Data analysis

To explore differences in the outcomes of interest across the sample, we divided the participants into three age groups based on the 33rd and 66th percentiles of the age distribution: younger children, middle-age, and older-age children. We then conducted separate one-way ANOVA tests for each interview outcome: total score, biophilia, and pro-environmental behaviors. The Shapiro–Wilk test indicated a statistically significant deviation from normality (*p* < 0.05). However, visual inspection of the Q-Q plot and histogram of residuals suggested that deviations were not extreme. Given our sample size, we proceeded with ANOVA, as mild violations of normality are generally acceptable as it has been shown that when Type I error occurs the F-test is still robust (Blanca et al., 2017). Levene’s test confirmed that the assumption of equal variances was met (*p* > 0.05), supporting the use of standard one-way ANOVA.

Finally, we conducted correlation analyses to examine relationships among key variables within each age group.

## Results

**Figure 1:**
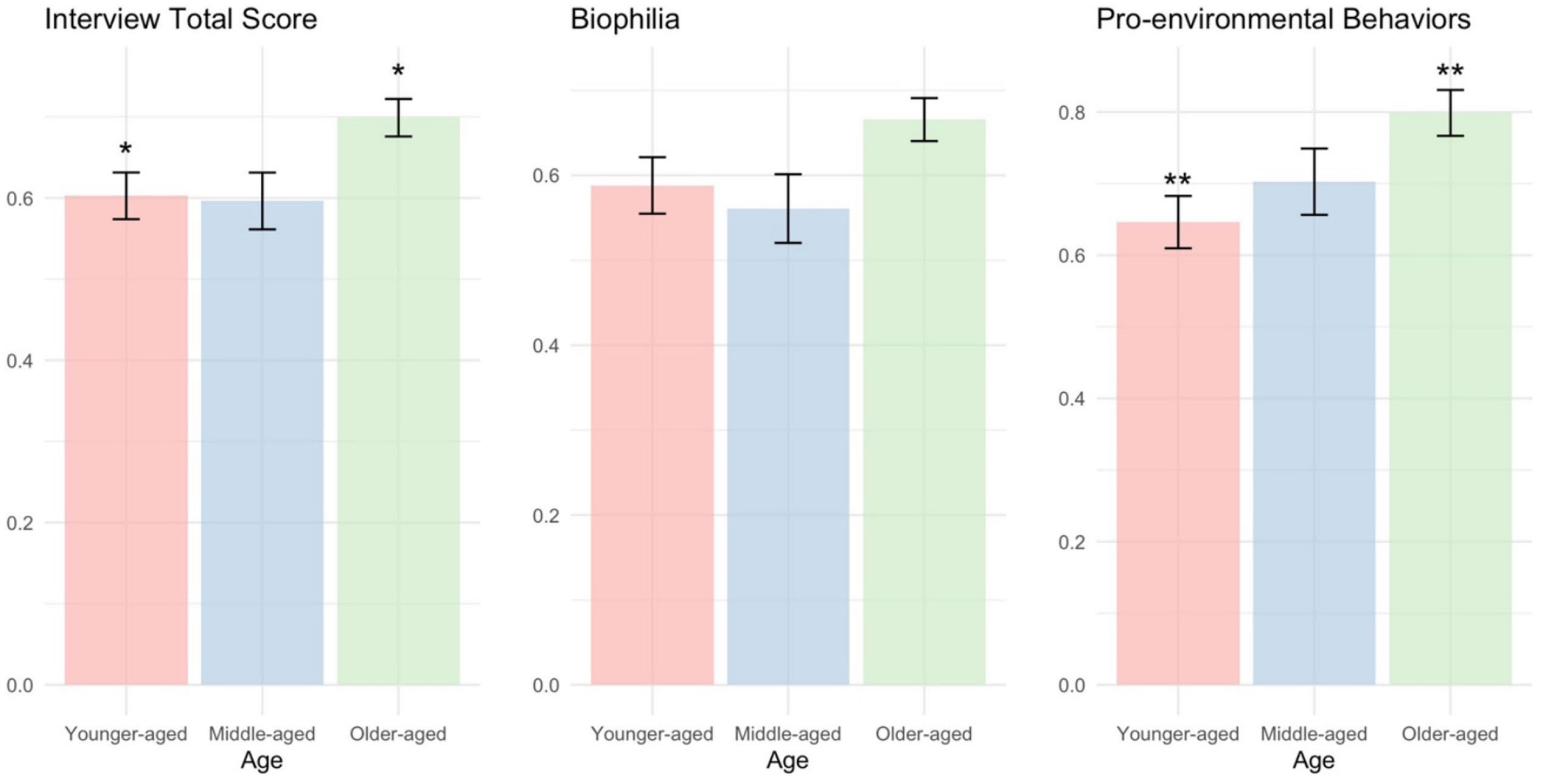
Bar graphs showing one-way ANOVA analysis. Bars represent mean scores, and error bars indicate standard errors (SE). Significant pairwise differences based on Tukey HSD post-hoc tests are marked with *p < 0.05 and **p < 0.01.

Results revealed significant differences in the total interview score across the age categories *F*_(2,166)_ = 5.404, *p* < 0.01. Post-hoc comparisons using the Tukey HSD test showed that older-age children scored significantly higher than middle-age (*p = 0*.02) and younger children (*p* = 0.01) (Figure 1). A separate ANOVA indicated a significant effect of age in biophilia, *F*_(2, 165)_ = 3.37, *p* < 0.05. However, post-hoc tests revealed no statistically significant differences among the groups. The results for pro-environmental behaviors showed significant group differences, *F*_(2, 165)_ = 5.13, *p* < 0.01. Subsequent pairwise comparisons revealed that older-aged children engaged in significantly more pro-environmental behaviors than younger children (*p* = 0.00).

No differences were found based on children’s gender (*F*_(1,172)_ = 0.07, *p* = 0.79) nor between schools (*F*_(1, 184)_ = 0.95, *p* = 0.47).

Considering the ANOVA results, we examined the correlations among variables for the three different age groups of children. For younger children (Table 1), the total score of the interview demonstrated significant positive correlations with their connectedness with nature as valuated by their parents (*r* = 0.29, *p* < 0.05). Children’s nature connectedness was also correlated with biophilia (*r* = 0.26, *p* < 0.05), nature exposure (*r* = 0.30, *p* < 0.05) and marginally with parents’ pro-environmental behaviors (*r* = 0.26, *p* = 0.06).

**Table 1:** Correlations among variables for younger children.

	1	2	3	4	5
1. Interview: total score	–				
2. Interview: biophilia	0.96***	–			
3. Interview: eco-friendly behavior	0.55***	0.27*	–		
4. Children’s connectedness with nature ^a^	0.29*	0.26*	0.21	–	
5. Parents’ eco-friendly behavior	0.12	0.07	0.18	0.26	–
6. Nature exposure ^a^	0.01	0.04	−0.11	0.30*	0.11

*p < 0.05; **p < 0.01; ***p < 0.001.

a Indirect measure filled in by parents.

For middle-age children (Table 2) and older children (Table 3), the correlations between the interview and the other variables are not statistically significant. Parents’ pro-environmental behavior correlates significantly with children’s connectedness with nature in middle-age children (*r* = 0.53, *p* < 0.001) and older children (*r* = 0.27, *p* < 0.05).

**Table 2:** Correlations among variables for middle age children.

	1	2	3	4	5
1. Interview: total score	–				
2. Interview: biophilia	0.95***	–			
3. Interview: eco-friendly behavior	0.54***	0.24	–		
4. Children’s connectedness with nature ^a^	−0.15	−0.12	−0.15	–	
5. Parents’ eco-friendly behavior	−0.06	−0.03	−0.15	0.53***	–
6. Nature exposure ^a^	−0.30	−0.28	−0.24	0.23	0.01

*p < 0.05; **p < 0.01; ***p < 0.001.

a Indirect measure filled in by parents.

**Table 3:** Correlations among variables for old-age children.

	1	2	3	4	5
1. Interview: total score	–				
2. Interview: biophilia	0.95***	–			
3. Interview: eco-friendly behavior	0.64***	0.36**	–		
4. Children’s connectedness with nature ^a^	0.12	0.07	0.17	–	
5. Parents’ eco-friendly behavior	0.08	0.12	−0.05	0.27*	–
6. Nature exposure ^a^	0.01	−0.01	0.06	0.32**	0.08

*p < 0.05; **p < 0.01; ***p < 0.001.

a Indirect measure filled in by parents.

Lastly, there is a significant correlation between children’s connectedness with nature and nature exposure in older age children (*r* = 0.32, *p* < 0.01).

## Discussion

These preliminary findings provide insights into preschool children’s biophilia and pro-environmental behaviors, as well as the role of age, nature exposure, connectedness to nature, and parents’ pro-environmental behaviors. The results, gained through a direct interview to the children and a questionnaire to parents offer an opportunity for some methodological reflections.

We found that biophilia scores do not significantly change across the three age groups, consistent with the idea that biophilia is an innate trait grounded in our species’ biology (Fromm, 1973; Wilson, 1993; Barbiero and Berto, 2021). On the contrary, the scores related to pro-environmental behaviors change across age groups, indicating that older children engage in significantly more pro-environmental behaviors than younger children. This variation in pro-environmental behavior scores across age groups underscores the role of education and socialization in fostering ecological habits. Older children demonstrating significantly more pro-environmental behaviors than younger children suggest that these behaviors might be the effect of education on how to adopt pro-environmental behaviors that the children receive in the family and at preschool. Studies have shown that environmental education and parental modeling play pivotal roles in developing sustainable behaviors in children (e.g., Collado et al., 2017). For instance, during the preschool years, children are often exposed to activities and curricula that emphasize recycling, conservation, and respect for nature, which could account for the progressive increase in pro-environmental behavior with age. Furthermore, the family environment serves as a critical context for nurturing pro-environmental behaviors. Research indicates that parental attitudes and practices regarding environmental responsibility significantly shape children’s behavior (Giancola et al., 2024). Features of the cognitive development of the children involved in the study could also explain these results. In fact, the adoption of pro-environmental behaviors can be linked to the children’s moral and social development and to a series of internal factors such as knowledge, intentions, and attitudes (Liu and Green, 2024). Very young children may have a limited knowledge of the systemic nature of the human - nature relationship to support their environmental moral reasoning and pro-environmental behaviors. Even if signs of an ecological awareness can be found even in young children, the developmental pattern of environmental moral reasoning is still unclear (Krettenauer, 2017). The younger children in our study may still have an egocentric perspective leading to a weaker tendency to protect the environment through specific behaviors.

The findings regarding the relationship between children’s biophilia and pro-environmental behaviors as measured through the interview, and the other variables assessed via parental reports present a mixed and more complex picture. Only for children in the youngest group of age biophilia scores are associated with their nature connectedness and nature exposure aligning with existing literature (Ahmetoglu, 2017; Mockovcáková and Barrable, 2024). For the middle-aged and older-age groups, significant correlations are found exclusively among variables assessed through parents’ reports, namely children’s connectedness to nature, parents’ pro-environmental behaviors, and exposure to nature. This last result is consistent with the literature showing that living in a place with high naturalness is linked to higher levels of connectedness to nature in school aged children (Barbiero and Berto, 2021; Stocco et al., 2023).

These two sets of findings seem to give reason to raise some reflections about the use of direct and indirect measure of children’s characteristics. In studying young children’s development and behavior the use of indirect measure gives the opportunity of collecting data on variables that would be too difficult and resource-consuming to collect directly from the children. Some examples of this are the evaluation of language competence or of executive functions during infancy and preschool age, where the use of parental reports provide the possibility of collecting reliable and valid data in a parsimonious way without requiring hours and hours of observation or to involve the child in challenging tasks (Garro, 2016). If in the case of early language competence evaluation there are checklists able to identify typical and atypical patterns of language development in children with good reliability and validity (e.g., the Italian version of MacArthur-Bates Communicative Development Inventory (MB-CDI), Caselli et al., 2015), on the other hand, the evaluation of children’s executive functions with direct or indirect tools seem to give different results (Willoughby and Hudson, 2021), probably because they measure different processes; furthermore, different witnesses (e.g., parents or teachers) of child’s executive functions may give different evaluations probably linked to the context-specific nature of the executive function skills (Wallisch et al., 2018; Spataro et al., 2024).

To our knowledge no specific research has been done to investigate the relationship between direct and indirect measures of biophilia and connectedness to nature. Our preliminary findings suggest that incorporating both types of measurements may provide a more comprehensive understanding of these constructs in preschool children. However, further research is needed to validate this approach and explore the interplay between these measurement methods. In fact, the lack of association between biophilia scores derived from children’s interview and connectedness to nature as reported by parents may stem from several factors. One possibility could be the differences in the item structure of the two instruments or in the core definition of the two factors. Alternatively, this discrepancy could reflect differing perspectives on the child’s behavior, with children and parents interpreting and reporting behaviors in distinct ways.

In the first case a reflection on the conceptual meaning of biophilia and of connectedness to nature in children will be necessary, to critically examine and refine the theoretical definitions of these constructs as they apply to children. On the other hand, if the issue arises from different viewpoints, future research should focus on exploring the implication of the two types of measures in relation to other factors and variables, such as experiences in nature, pro-environmental behavior, and related constructs.

## Conclusion

The findings, while preliminary, highlight the complexity of assessing biophilia, connectedness to nature and pro-environmental behaviors in preschool-aged children, particularly when combining direct (child interviews) and indirect (parental reports) measures. Each method offers unique contributions, with direct measures capturing children’s self-perceptions and indirect measures reflecting external observations and surely need more research to understand the intricated relationships between these constructs. However, these insights have valuable implications for designing interventions and educational programs aimed at fostering biophilia and pro-environmental behaviors starting from preschool.

Some limitations must be acknowledged. First, parental reports may be subject to bias due to subjective perceptions or social desirability. Second, as a preliminary study, the sample size and demographic characteristics limit the generalizability of the findings, although this was not the study’s aim. Third, differences in the structure and focus of the measurement instruments may have influenced the results. Finally, the cross-sectional design prevents to draw causal inferences, highlighting the need for longitudinal and experimental research to build on these findings.

Future studies should refine the conceptualization of biophilia and connectedness to nature in children, exploring their developmental trajectories and relationships over time. Research should also explore how these measures interact with other variables to offer a more comprehensive understanding of the factors influencing children’s environmental attitudes and behaviors.

## Data Availability

The raw data supporting the conclusions of this article will be made available by the authors, without undue reservation.

## References

[ref1] Ahmetoglu E. (2017). The contributions of familial and environmental factors to children’s connection with nature and outdoor activities. Early Child Dev. Care 189, 233–243. doi: 10.1080/03004430.2017.1314273, PMID: 40101104

[ref2] Bagot K. L. Allen F. C. L. Toukhsati S. (2015). Perceived restorativeness of children’s school playground environments: nature, playground features and play period experiences. J. Environ. Psychol. 41, 1–9. doi: 10.1016/j.jenvp.2014.11.005

[ref3] Barbiero G. Berto R. (2021). Biophilia as evolutionary adaptation: an onto-and phylogenetic framework for biophilic design. Front. Psychol. 12:700709. doi: 10.3389/fpsyg.2021.700709, PMID: 34367025 PMC8334556

[ref4] Beery T. Fridberg M. Præstholm S. Uhnger Wünsche T. Bølling M. (2024). Connectedness to nature: a tale of three scales. Environ. Educ. Res. 30, 1783–1805. doi: 10.1080/13504622.2024.2320342

[ref5] Berto R. Maculan N. Barbiero G. (2020). Does sustainability address perceived restoration? An exploratory study on Biosphera 2.0, a net zero energy house. Visions Sustain. 13, 17–30. doi: 10.13135/2384-8677/4181

[ref6] Blanca M. J. Alarcón R. Arnau J. Bono R. Bendayan R. (2017). Non-normal data: is ANOVA still a valid option? Psicothema 4, 552–557. doi: 10.7334/psicothema2016.383, PMID: 29048317

[ref7] Carrus G. Passiatore Y. Pirchio S. Scopelliti M. (2015a). Contact with nature in educational settings might help cognitive functioning and promote positive social behaviour. PsyEcology 6, 191–212. doi: 10.1080/21711976.2015.1026079

[ref8] Carrus G. Pirchio S. Tiberio L. (2020). Transitions to sustainability, lifestyles changes and human well-being: cultural, environmental and political challenges (Transiciones hacia la sostenibilidad, cambios de estilos de vida y bienestar humano: desafíos culturales, medioambientales y políticos). PsyEcology 11, 163–169. doi: 10.1080/21711976.2020.1734411

[ref9] Carrus G. Scopelliti M. Lafortezza R. Colangelo G. Ferrini F. Salbitano F.. (2015b). Go greener, feel better? The positive effects of biodiversity on the well-being of individuals visiting urban and peri-urban green areas. Landsc. Urban Plan. 134, 221–228. doi: 10.1016/j.landurbplan.2014.10.022

[ref10] Carrus G. Scopelliti M. Panno A. Lafortezza R. Colangelo G. Pirchio S.. (2017). A different way to stay in touch with ‘urban nature’: the perceived restorative qualities of botanical gardens. Front. Psychol. 8:914. doi: 10.3389/fpsyg.2017.00914, PMID: 28620335 PMC5450850

[ref11] Caselli M. C. Bello A. Rinaldi P. Stefanini S. Pasqualetti P. (2015). Il Primo Vocabolario del Bambino: Gesti, Parole e Frasi. Valori di riferimento fra 8 e 36 mesi delle Forme complete e delle Forme brevi del questionario MacArthur-Bates CDI: Valori di riferimento fra 8 e 36 mesi delle Forme complete e delle Forme brevi del questionario MacArthur-Bates CDI. Rome: FrancoAngeli.

[ref12] Cerina V. Fornara F. (2011). The psychological determinants of attitudes toward relocation in the elderly: a survey study in urban and rural environments. PsyEcology 2, 335–348. doi: 10.1174/217119711797877744

[ref13] Collado S. Evans G. W. Sorrel M. A. (2017). The role of parents and best friends in children’s pro-environmentalism: differences according to age and gender. J. Environ. Psychol. 54, 27–37. doi: 10.1016/j.jenvp.2017.09.007, PMID: 40104671

[ref14] Collado S. Staats H. (2016). Contact with nature and children’s restorative experiences: an eye to the future. Front. Psychol. 7:1885. doi: 10.3389/fpsyg.2016.01885 27965616 PMC5126106

[ref15] Federico F. (2020). Natural environment and social relationship in the development of attentional network. Front. Psychol. 11:1345. doi: 10.3389/fpsyg.2020.01345, PMID: 32670162 PMC7332839

[ref16] Fromm E. (1973). The Anatomy of Human Destructiveness. New York: Fawcett Crest.

[ref17] Garro A. (2016). Early childhood assessment in school and clinical child psychology. New York: Springer.

[ref18] Giancola M. Pino M. C. Zacheo C. Sannino M. D’Amico S. (2024). The intergenerational transmission of pro-environmental behaviours: the role of moral judgment in primary school-age children. Soc. Sci. 13:318. doi: 10.3390/socsci13060318

[ref19] Hartig T. (2004). “Restorative environments” in Encyclopedia of applied psychology. ed. Spielberger C. (New York, NY: Academic Press/Elsevier), 273–279.

[ref20] Hartig T. van den Berg A. E. Hagerhall C. M. Tomalak M. Bauer N. Hansmann R.. (2011). “Health benefits of nature experience: psychological, social and cultural processes” in Forests, trees and human health. ed. Nilsson K. (Dordrecht: Springer), 127–168.

[ref21] Harvey D. J. Montgomery L. N. Harvey H. Hall F. Gange A. C. Watling D. (2020). Psychological benefits of a biodiversity-focussed outdoor learning program for primary school children. J. Environ. Psychol. 67:101381. doi: 10.1016/j.jenvp.2019.101381

[ref22] Johnson S. A. Snow S. Lawrence M. A. Rainham D. G. C. (2019). Quasi-randomized trial of contact with nature and effects on attention in children. Front. Psychol. 10:2652. doi: 10.3389/fpsyg.2019.02652, PMID: 31866892 PMC6907393

[ref23] Kabisch N. van den Bosch M. Lafortezza R. (2017). The health benefits of nature-based solutions to urbanization challenges for children and the elderly–a systematic review. Environ. Res. 159, 362–373. doi: 10.1016/j.envres.2017.08.004, PMID: 28843167

[ref24] Kaplan S. Berman M. G. (2010). Directed attention as a common resource for executive functioning and self-regulation. Perspect. Psychol. Sci. 5, 43–57. doi: 10.1177/1745691609356784, PMID: 26162062

[ref25] Kellert S. R. Wilson E. O. (1993). The biophilia hypothesis. Washington, DC: Island Press.

[ref26] Krettenauer T. (2017). Pro-environmental behavior and adolescent moral development. J. Res. Adolesc. 27, 581–593. doi: 10.1111/jora.12300, PMID: 28776840

[ref27] Kumawat P. Pronello C. (2021). Validating Italian general ecological behaviour questionnaire of travellers using dichotomous Rasch model. Sustain. For. 13:11976. doi: 10.3390/su132111976

[ref28] Lafortezza R. Carrus G. Sanesi G. Davies C. (2009). Benefits and well- being perceived by people visiting green spaces in periods of heat stress. Urban Forestry Urban Greening 8, 97–108. doi: 10.1016/j.ufug.2009.02.003

[ref29] Liefländer A. K. Fröhlich G. Bogner F. X. Schultz P. W. (2013). Promoting connectedness with nature through environmental education. Environ. Educ. Res. 19, 370–384. doi: 10.1080/13504622.2012.697545

[ref30] Liu J. Green R. J. (2024). Children’s pro-environmental behaviour: a systematic review of the literature. Resour. Conserv. Recycl. 205:107524. doi: 10.1016/j.resconrec.2024.107524

[ref31] Louv R. (2005). Last child in the woods: saving our children from nature deficit disorder. New York: Workman Publishing Company.

[ref32] Maricchiolo F. Mosca O. Lauriola M. Krys K. (2020). The role of urbanization of place of living in the relation between individual features and happiness (El papel del desarrollo urbanístico del lugar de residencia en la relación entre las características individuales y la felicidad). PsyEcology 11, 232–259. doi: 10.1080/21711976.2020.1734399

[ref33] Marte E. Calumpit A. de Sá Bessa B. Toledo A. Fadda R. Skoler T. (2020). Testing reliability of Biophilic design matrix within urban residential playrooms. Front. Psychol. 11:570099. doi: 10.3389/fpsyg.2020.570099, PMID: 33362635 PMC7756148

[ref34] Mockovcáková A. Barrable A. (2024). Factors associated with nature connection in children: a review, synthesis, and implications for practice within environmental education and beyond. Int. J. Early Childhood Environ. Educ. 11, 26–42.

[ref35] Otto S. Pensini P. (2017). Nature-based environmental education of children: environmental knowledge and connectedness to nature, together, are related to ecological behaviour. Global Environ. Change 47, 88–94. doi: 10.1016/j.gloenvcha.2017.09.009

[ref36] Passafaro P. Carrus G. Pirchio S. (2010). I Bambini e L’Ecologia, Aspetti Psicologici Dell’Educazione Ambientale. Rome: Carrocci editore.

[ref37] Pirchio S. Passiatore Y. Panno A. Cipparone M. Carrus G. (2021). The effects of contact with nature during outdoor environmental education on students’ wellbeing, connectedness to nature and pro-sociality. Front. Psychol. 12:648458. doi: 10.3389/fpsyg.2021.648458, PMID: 34017288 PMC8129515

[ref38] Rice C. S. Torquati J. C. (2013). Assessing connections between young children's affinity for nature and their experiences in natural outdoor settings in preschools. Child. Youth Environ. 23, 78–102. doi: 10.1353/cye.2013.0051

[ref39] Sobko T. Jia Z. Brown G. (2018). Measuring connectedness to nature in preschool children in an urban setting and its relation to psychological functioning. PLoS One 13:e0207057, PMID: 30496300 10.1371/journal.pone.0207057 PMC6264829

[ref40] Spataro P. Morelli M. Pirchio S. Costa S. Longobardi E. (2024). Exploring the relations of executive functions with emotional, linguistic, and cognitive skills in preschool children: parents vs. teachers reports. Eur. J. Psychol. Educ. 39, 1045–1067. doi: 10.1007/s10212-023-00749-7

[ref41] Stocco A. Tabacchi C. Barbiero G. Pranovi F. (2023). The influence of naturalness of the landscape structure on children’s connectedness to nature in North-Eastern Italy. One Ecosystem 8:e111973. doi: 10.3897/oneeco.8.e111973

[ref42] Tarman İ. Erbay F. Durmusoglu-Saltali N. (2023). The relationship between biophilia levels of preschoolers and social-emotional well-being and psychological resilience in the context of age and gender. Early Child Dev. Care 193, 1041–1054. doi: 10.1080/03004430.2023.2216892

[ref43] Varela-Candamio L. Novo-Corti I. García-Álvarez M. T. (2018). The importance of environmental education in the determinants of green behavior: a meta-analysis approach. J. Clean. Prod. 170, 1565–1578. doi: 10.1016/j.jclepro.2017.09.214

[ref44] Wallisch A. Little L. M. Dean E. Dunn W. (2018). Executive function measures for children: a scoping review of ecological validity. OTJR Occup. Particip. Health 38, 6–14. doi: 10.1177/1539449217727118 28891377

[ref45] Wassenberg C. L. Goldenberg M. A. Soule K. E. (2015). Benefits of botanical garden visitation: a means-end study. Urban Forestry Urban Green 14, 148–155. doi: 10.1016/j.ufug.2015.01.002

[ref46] Willoughby M. Hudson K. (2021). “Current issues in the conceptualization and measurement of executive function skills” in Executive functions and writing. eds. Limpo T. Olive T. (Oxford: Oxford University Press), 17–37.

[ref47] Wilson E. O. (1993). “Biophilia and the conservation ethic” in The biophilia hypothesis. eds. Kellert S. R. Wilson E. O. (Washington, DC: Island Press), 31–41.

[ref48] Wu H. Ji R. Jin H. (2023). Parental factors affecting children’s nature connectedness. J. Environ. Psychol. 87:101977. doi: 10.1016/j.jenvp.2023.101977

[ref49] Yilmaz S. (2017). Investigation of five-year-old preschool children’s biophilia and children’s and their mothers’ outdoor setting preferences. Ph.D. thesis submitted to Middle. Ankara, Turkey: East Technical University.

