# Supplementary material for: Biophilia in Italian preschool children: preliminary findings
Source: Front Psychol. 2025 Apr 9;16:1567848. doi: 10.3389/fpsyg.2025.1567848 (PMC12014690; doi:10.3389/fpsyg.2025.1567848)
Supplement: Supplementary file 1 [file Data_Sheet_1.pdf]

Dipartimento
di Psicologia Dinamica
Clinica e Salute

Sapienza
Università di Roma

Esperienze di contatto con la natura in età prescolare

Sapienza Università di Roma – Dipartimento di Psicologia Dinamica, Clinica e Salute

Responsabili del progetto: Prof.ssa Rosa Ferri e Prof.ssa Sabine Pirchio

L'obiettivo della ricerca è valutare gli effetti dell'*outdoor education* in età prescolare sul senso di connessione con la natura, sui comportamenti ecologici, sul comportamento adattivo, pro-sociale e di autocontrollo in bambini e bambine che frequentano la scuola dell'infanzia indagando nello specifico l'effetto di esperienze di esposizione alla natura.

Prima di cominciare le chiediamo di creare un codice seguendo le indicazioni sottostanti:

- prime 3 lettere del nome di suo/sua figlio/a,
- prime 3 lettere del cognome di suo/sua figlio/a,
- numero del giorno di nascita di suo/a figlio/a,
- la lettera del genere biologico di suo/sua figlio/a (F per femmina, M per maschio)

Es. se la bambina si chiama Giulia Rossi ed è nata il 07 maggio 2019, il codice sarà: GIUROS07F

CODICE: ____________________

Nelle pagine che seguono trova:
- il questionario socio-anagrafico relativo alla sua famiglia
- il questionario sul suo comportamento ecologico
- il questionario sul senso di connessione con la natura di suo/a figlio/a

DIPARTIMENTO
DI PSICOLOGIA DINAMICA
CLINICA E SALUTE

SAPIENZA
UNIVERSITÀ DI ROMA

Questionario socio-anagrafico relativo alla sua famiglia

INFORMAZIONI SUL BAMBINO

GENERE del bambino □ M □ F □ Altro

ETÀ in mesi ________

Data di nascita del bambino ____________

Ordine di nascita □ primogenito □ secondogenito □ terzogenito □ altro:_____________

Suo/a figlio/a è nato prematuro? □ No □ Si

Se sì, di quante settimane è nato prematuro? _______

Peso di suo figlio alla nascita: ____________ grammi

Ci sono state complicazioni alla nascita? □ No □ Si □ Solo sospettato

Suo figlio presenta ritardo o difficoltà relative al linguaggio? □ No □ Si □ Solo sospettato

Problemi relativi all'udito? □ No □ Si □ Solo sospettato

Problemi relativi allo sviluppo? □ No □ Si □ Solo sospettato

Altri problemi di salute? □ No □ Si □ Solo sospettato

Se ha risposto sì ad una delle precedenti domande, fornisca una descrizione:

____

____

____

____

Quante persone compongono il nucleo familiare? _____________

Quanti fratelli/sorelle ha suo figlio/sua figlia?___________

INFORMAZIONI SU CHI COMPILA IL QUESTIONARIO

Relazione tra il bambino e la persona che compila il questionario

□ Madre □ Padre □ Altro (specificare __________)

Genere: □ M □ F **Età:**_____

Titolo di studio:

□ Licenza Elementare
□ Diploma di Scuola Media Inferiore
□ Diploma di Scuola Media Superiore
□ Laurea
□ Corsi post-universitari

Professione/impiego/occupazione: ………………………………………

Indicativamente, in quale categoria di reddito si colloca il suo nucleo familiare?

□ 0-15.000 euro/annui
□ 15.001-28.000 euro/annui
□ 28.001-55.000 euro/annui
□ 55.001-75.000 euro/annui
□ oltre 75.000 euro/annui
□ Preferisco non rispondere

Lei attualmente è:

□ single
□ fidanzato/a
□ convivente
□ sposato/a
□ separato/a - divorziato/a
□ vedovo/a

Dipartimento
di Psicologia Dinamica
Clinica e Salute

Sapienza
Università di Roma

Questionario sul comportamento ecologico di chi compila il questionario

Le chiediamo di indicare il suo grado di accordo con le seguenti affermazioni riferite al suo comportamento. Indichi per ogni affermazione quanto essa corrisponda al suo comportamento su una scala da **1 (completamente in disaccordo)** a **6 (completamente d'accordo).**

	**completamente in disaccordo**					**completamente d'accordo**
Capita che dia l'elemosina ai senza-tetto lungo la strada	1	2	3	4	5	6
Di tanto in tanto dono dei soldi per beneficenza	1	2	3	4	5	6
Se una persona anziana o disabile entra in un bus affollato, offro a lei il mio posto a sedere	1	2	3	4	5	6
Se io fossi un datore di lavoro, avrei problemi ad assumere una persona con precedenti penali	1	2	3	4	5	6
Ogni tanto prendo i mezzi pubblici senza pagare il biglietto	1	2	3	4	5	6
Getto nell'indifferenziato le batterie esaurite	1	2	3	4	5	6
Separo il vetro dagli altri rifiuti per riciclarlo	1	2	3	4	5	6
Spengo il riscaldamento durante la notte	1	2	3	4	5	6
Aspetto di potere fare un pieno carico prima di fare il bucato con la lavatrice	1	2	3	4	5	6
In inverno, lascio la finestra aperta a lungo per fare entrare aria fresca e pulita	1	2	3	4	5	6
Per il mio bucato uso l'ammorbidente	1	2	3	4	5	6
Cerco sempre di comprare prodotti biologici	1	2	3	4	5	6
Ogni tanto, vendo le cose che non uso più	1	2	3	4	5	6
Ogni tanto, compro cose di seconda mano	1	2	3	4	5	6
Ogni tanto, regalo cose che non uso più	1	2	3	4	5	6
Ogni tanto, presto le cose che uso poco	1	2	3	4	5	6
Mangio meno carne rispetto agli scorsi anni	1	2	3	4	5	6
Riuso la borsa della spesa per le volte successive	1	2	3	4	5	6
Ogni tanto compro delle bibite in lattina	1	2	3	4	5	6
Spesso parlo con i miei amici dei problemi legati all'ambiente	1	2	3	4	5	6
Sono attiva/o in un'organizzazione ambientalista	1	2	3	4	5	6
Nel passato, mi è capitato di fare notare a qualcuno il suo comportamento non ecologico	1	2	3	4	5	6
Ogni tanto sostengo economicamente le organizzazioni ambientali	1	2	3	4	5	6
Boicotto le compagnie che usano i pesticidi o gli OGM	1	2	3	4	5	6
Di solito, non guido la mia automobile in città	1	2	3	4	5	6
In generale, in autostrada mantengo una velocità inferiore ai 100 km all'ora	1	2	3	4	5	6

Dipartimento
di Psicologia Dinamica
Clinica e Salute

Sapienza
Università di Roma

Questionario sulla connessione con la natura di suo/a figlio/a

Le chiediamo di indicare il suo grado di accordo con le seguenti affermazioni. Indichi per ogni affermazione quanto essa descriva suo/a figlio/a, su una scala da ***1 (fortemente in disaccordo)*** a ***5 (fortemente d'accordo)***.

	**fortemente in disaccordo**				**fortemente d'accordo**
A mio figlio piace sentire i diversi suoni della natura	1	2	3	4	5
A mio figlio piace vedere i fiori di campo nella natura	1	2	3	4	5
Stare nella natura fa sentire mio figlio tranquillo	1	2	3	4	5
A mio figlio piace fare giardinaggio e piantare	1	2	3	4	5
Mio figlio si diverte a collezionare pietre e conchiglie	1	2	3	4	5
A mio figlio piace toccare animali e piante	1	2	3	4	5
Mio figlio si sente triste quando gli animali selvatici sono feriti	1	2	3	4	5
Mio figlio è angosciato quando vede animali che vengono feriti	1	2	3	4	5
Mio figlio è affranto quando gli animali muoiono	1	2	3	4	5
Mio figlio pensa che raccogliere la spazzatura da terra possa aiutare la natura	1	2	3	4	5
Mio figlio tratta piante, animali e insetti con cura	1	2	3	4	5
Mio figlio tratta piante, animali e insetti con cura	1	2	3	4	5
A mio figlio piace riciclare carta e bottiglie	1	2	3	4	5
Mio figlio nota animali selvatici ovunque si trovi	1	2	3	4	5
Mio figlio sceglie letture che parlano di piante e animali	1	2	3	4	5
Mio figlio sente la differenza tra ambiente esterno e interno	1	2	3	4	5
Mio figlio sente uccelli e altri suoni della natura	1	2	3	4	5

Ripensando all'ultimo mese con quale frequenza suo figlio ha frequentato i seguenti luoghi escludendo le attività scolastiche? Risponda mettendo una croce nella casella appropriata.

	**mai**	**meno di 3 volte**	**Tra 3 e 8 volte**	**3 volte a settimana**	**Più di 3 volte a settimana**
Parco pubblico					
Spiaggia					
Area naturale di campagna					
Area naturale di montagna					

Ripensando all'ultimo mese e ai luoghi che suo figlio ha frequentato al di fuori delle attività scolastiche, in media per quanto tempo li ha frequentati **OGNI** volta? Risponda mettendo una croce nella casella appropriata

	**Meno di 30 minuti**	**Tra 30 e 60 minuti**	**Tra 1 e 2 ore**	**Mezza giornata**	**Tutta la giornata**
Parco pubblico					
Spiaggia					
Area naturale di campagna					
Area naturale di montagna					

La compilazione della sua parte di questionario è conclusa!

Grazie della sua collaborazione!
