# Supplementary material for: Biophilia in Italian preschool children: preliminary findings
Source: Front Psychol. 2025 Apr 9;16:1567848. doi: 10.3389/fpsyg.2025.1567848 (PMC12014690; doi:10.3389/fpsyg.2025.1567848)
Supplement: Supplementary file 2 [file Data_Sheet_2.pdf]

Protocollo per l'intervista su Biofilia e Comportamenti ecologici

Consegna
"Ciao, ti presento due bimbi/e"
"Lui/lei è Luca/Chiara (mostrare il primo pupazzetto) e lui è Paolo/Nina (mostrare il secondo pupazzetto)."
"a Luca e Paolo / Chiara e Nina piacciono cose diverse e si divertono con giochi diversi. Mi dici tu quali cose e giochi preferisci?"
"Per esempio, a Luca/Chiara piace il gelato, a Paolo /Nina piace il gelato. Tu sei più come Luca/Chiara o come Paolo /Nina?" "Come Luca" "Ah allora ti piace il gelato"
Accertarsi che il bambino abbia compreso la logica dell'intervista, se necessario fare altre prove.

Protocollo

Domanda		Risposta
a Luca/Chiara piace il gelato	a Paolo /Nina non piace tanto il gelato	*Luca*
a Luca/Chiara piace giocare fuori	a Paolo /Nina piace giocare dentro	
a Paolo /Nina piace scavare per cercare vermi	a Luca/Chiara non piacciono i vermi	
a Luca/Chiara piace saltare nelle pozzanghere	a Paolo /Nina non piace infangarsi e bagnarsi	
a Paolo /Nina piace guardare gli uccelli	a Luca/Chiara non piace guardare gli uccelli	
a Luca/Chiara piace catturare insetti e guardarli	a Paolo /Nina piace calpestare gli insetti e ucciderli	
a Paolo /Nina piace guardare animali come scoiattoli e conigli	Luca/Chiara pensa che è noioso guardare gli animali	
a Luca/Chiara piace giocare in ruscelli e laghi	a Paolo /Nina non piace sporcarsi e bagnarsi	
a Paolo /Nina piace giocare con bastoncini, foglie e pigne	Luca/Chiara pensa che bastoncini, foglie e pigne sono sporchi	
a Luca/Chiara piace ascoltare gli uccelli cantare	Paolo /Nina pensa che è noioso ascoltare gli uccelli cantare	
a Paolo /Nina piace guardare le stelle e la luna di notte	Luca/Chiara di notte preferisce giocare dentro casa	
a Luca/Chiara piace imparare cose sugli animali selvatici	a Paolo /Nina non interessano gli animali selvatici	
a Paolo /Nina piace sdraiarsi in un prato a guardare le nuvole	Luca/Chiara preferisce sdraiarsi sul divano a guardare la tv	

Luca/Chiara chiude l'acqua quando si lava i denti	Paolo /Nina lascia l'acqua a aperta per tutto il tempo in cui si lava i denti	
Paolo /Nina spegne la luce quando esce da una stanza	Luca/Chiara lascia la luce accesa anche quando esce da una stanza	
Luca/Chiara sta attento a riciclare le cose che usa	Paolo /Nina butta via le cose che non usa più	
a Paolo /Nina dispiace quando si tagliano gli alberi	Luca/Chiara pensa che è giusto che si taglino gli alberi	
